# Plasma Chemerin Is Induced in Critically Ill Patients with Gram-Positive Infections

**DOI:** 10.3390/biomedicines11071779

**Published:** 2023-06-21

**Authors:** Pablo Amend, Patricia Mester, Stephan Schmid, Martina Müller, Christa Buechler, Vlad Pavel

**Affiliations:** Department of Internal Medicine I, Gastroenterology, Hepatology, Endocrinology, Rheumatology and Infectious Diseases, University Hospital Regensburg, 93053 Regensburg, Germany; pablo.amend@stud.uni-regensburg.de (P.A.); patricia.mester@klinik.uni-regensburg.de (P.M.); stephan.schmid@klinik.uni-regensburg.de (S.S.); martina.mueller-schilling@klinik.uni-regensburg.de (M.M.); vlad.pavel@klinik.uni-regensburg.de (V.P.)

**Keywords:** COVID-19, C-reactive protein, sepsis, bacterial infection, sepsis biomarker, Gram-positive bacteria

## Abstract

Chemerin is a chemoattractant protein abundantly expressed in hepatocytes. Chemerin exerts pro- and anti-inflammatory effects and acts as a pro-resolving protein. Chemerin levels are low in patients with liver cirrhosis and are increased in sepsis. The aim of this study was to identify associations between plasma chemerin levels and underlying diseases as well as causes of severe illness. The cohort included 32 patients with liver cirrhosis who had low systemic chemerin, and who were not considered for further evaluation. Plasma chemerin levels were similar between the 27 patients with systemic inflammatory response syndrome (SIRS), the 34 patients with sepsis and the 63 patients with septic shock. Chemerin in plasma correlated with C-reactive protein and leukocyte count but not with procalcitonin, a clinical marker of bacterial infection. Plasma chemerin did not differ among patients with and without ventilation and patients with and without dialysis. Vasopressor therapy was not associated with altered plasma chemerin levels. Infection with severe acute respiratory syndrome coronavirus 2 had no effect on plasma chemerin levels. Baseline levels of plasma chemerin could not discriminate between survivors and non-survivors. Notably, Gram-positive infection was associated with higher chemerin levels. In summary, the current study suggests that plasma chemerin might serve as an early biomarker for the diagnosis of Gram-positive infections in patients with sepsis.

## 1. Introduction

Sepsis is a state of high-grade systemic inflammation in response to bacterial, fungal, or viral infections [1,2]. In about a third of the patients, an infectious pathogen is not detectable [3,4]. Early start of therapy is associated with a lower risk of death and management of sepsis involves antimicrobial treatment, fluid replacement and vasopressor therapy [2]. Therapeutic strategies targeting endotoxins such as lipopolysaccharide and the cytokine tumor necrosis factor showed no change in overall survival. Corticosteroids do not offer any survival advantage [2] and are not effective in patients with coronavirus disease 2019 (COVID-19) pneumonia [5]. Inflammation is essential for the activation of immune cells and the initiation of anti-inflammatory pathways that terminate the inflammatory process [3,4]. Patients treated with anti-inflammatory drugs in the immunosuppressive phase of sepsis have an increased risk of infections [4]. Hence, pathways that promote the resolution of inflammation are considered promising strategies for the treatment of sepsis [3,4].

Chemerin exerts pro- as well as anti-inflammatory activities and functions as a pro-resolving protein. Chemerin can activate the nuclear factor kappa B pathway in skeletal muscle cells and has the opposite effect on adipocytes [6,7,8,9,10].

Chemerin has to be processed by proteases in order to be biologically active. The C-terminal processing of chemerin leads to molecules with different biologic activities whose functional relevance still remains unclear. Chemerin isoforms exert opposing roles in inflammation. However, with commercial ELISAs, these variants can not be distinguished [7,10,11]. Chemerin recruits immune cells to sites of infections [12], and furthermore, exhibits bactericidal activity against *Escherichia coli*, *Candida albicans* and methicillin-resistant *Staphylococcus aureus* [13,14].

Chemerin expression in adipocytes is induced by inflammatory mediators such as lipopolysaccharide and tumor necrosis factor. The anti-inflammatory drugs tazarotene and dexamethasone upregulated chemerin in skin fibroblasts [6,10,15,16], further indicating a function of chemerin as an immunoregulatory molecule. 

Serum chemerin levels were consistently higher in patients with inflammatory diseases [17]. Accordingly, serum chemerin levels were significantly increased in sepsis patients in comparison to healthy controls [18,19]. Serum chemerin was induced in patients with septic shock as well as non-survivors of sepsis, illustrating associations of systemic chemerin with disease severity and outcome [18].

Severe acute respiratory syndrome coronavirus type 2 (SARS-CoV-2) infection may cause sepsis with acute respiratory distress syndrome (ARDS) as a frequent organ dysfunction [20]. COVID-19 patients were described to have lower serum chemerin in comparison to healthy controls [21]. In contrast, another study detected higher plasma chemerin levels in COVID-19 patients compared to healthy controls, with chemerin being even higher in non-surviving COVID-19 patients [22]. Finally, it was shown that serum chemerin levels were not associated with COVID-19 severity on the day of hospitalization. Chemerin levels of patients with severe COVID-19 declined during recovery [23]. 

Patients with pre-existing chronic liver diseases are at a higher risk for severe COVID-19 and sepsis [24,25]. Serum chemerin of patients with liver cirrhosis is low [26,27]. Serum levels of C-reactive protein (CRP) [28], the most widely used clinical marker for sepsis, are reduced in patients with liver cirrhosis, and this may limit the diagnostic utility of CRP [29,30]. Therefore, the association of circulating chemerin and CRP with sepsis severity is influenced by the underlying disease.

Procalcitonin and CRP are used for the diagnosis of sepsis and in assessing the severity of sepsis. These molecules cannot differentiate infectious from noninfectious diseases [28,31,32].

Identification of specific biomarkers for early diagnosis of bacterial and viral infections in sepsis would be of paramount importance. Current evidence suggests that underlying diseases such as liver cirrhosis influence circulating levels of commonly used clinical parameters [1,28,31]. The aim of our study was to evaluate associations of plasma chemerin with underlying diseases and causes of severe illness in a cohort of patients with systemic inflammatory response syndrome (SIRS), sepsis or septic shock.

## 2. Materials and Methods

### 2.1. Patients

Plasma of 156 patients was collected at the University Hospital of Regensburg from August 2018 until January 2023. The Sepsis-3 criteria were used, and accordingly, 40 patients were categorized as sepsis and 79 patients as septic shock [33]. The 37 patients who were admitted to the intensive care unit with suspected sepsis but did not develop sepsis during follow-up were categorized as SIRS. These patients had a sepsis-related organ failure assessment (SOFA) score < 2 and, according to the Sepsis-3 definition, do not have sepsis. This categorization is independent of infections [33,34]. Patients who had multi-resistant infections, human immunodeficiency virus infection or viral hepatitis were excluded. Controls were 10 healthy males and 6 females, the age of this cohort was 49 (26–75) years and did not significantly differ from the patients (*p* = 0.06).

### 2.2. Analysis of Chemerin

Blood samples from the patients were obtained at 12 to 24 h after admission to the intensive care unit. EDTA was used as an anticoagulant, and plasma was prepared. Plasma chemerin was determined by an enzyme-linked immunosorbent assay (ELISA) (R&D Systems, Wiesbaden, Nordenstadt, Germany; Cat # DY2324). Each sample was tested in duplicate and the mean value was used for further calculations. For chemerin ELISA, plasma was diluted 1:250 fold.

### 2.3. Analysis of C-Reactive Protein, Procalcitonin, IL-6 and Leukocyte Number

C-reactive protein was analyzed by a particle-enhanced immunoturbidimetric assay. Procalcitonin and IL-6 were analyzed using ElektroChemiLumineszenz ImmunoAssays. All assays were performed by using the Cobas Pro analyzer and the respective assays (Roche, Penzberg, Germany). Leukocytes were determined by an impedance/flow cytometry method using the Sysmex instrument (Sysmex Deutschland GmbH, Bornbarch, Germany). IL-6 levels were only analyzed in COVID-19. Laboratory parameters were determined at the Institute of Clinical Chemistry and Laboratory Medicine (University Hospital, Regensburg).

### 2.4. Microbiological Tests

Blood cultures in automatic systems (BD *BACTEC*™ FX Top-Unit; Becton Dickinson, Eysins, Switzerland), Gram staining and matrix-assisted laser desorption/ionization time-of-flight (MALDI-TOF) mass spectrometry (Bruker Microflex LT; Bruker, Hamburg, Germany) were used in the diagnosis of bacterial infections. These diagnostic tests were performed at the Institute of Clinical Microbiology and Hygiene, University Hospital Regensburg, and have been described in more detail [35]. *Escherichia coli*, *Enterococcus faecalis*, *Staphylococcus aureus* and *Staphylococcus epidermidis* are common pathogenic microorganisms in sepsis [36], and were detected in the blood of our patients. 

### 2.5. Statistical Analysis

Data are shown as boxplots, and the minimum value, the maximum value, the median and the first and third quartiles are displayed. Circles and asterisks mark outliers. Outliers that are more than 1.5× interquartile range below the lower quartile or above the upper quartile are represented by circles. Values that are more than 3.0× interquartile range below the lower quartile or above the upper quartile are represented by asterisks. Data are given as median, minimum, and maximum values. The Chi-square test (Ms Excel), the non-parametric Mann–Whitney U test, Kruskal–Wallis test as well as Spearman’s correlation were used for analysis (IBM SPSS Statistics 26.0 program). A value of *p* < 0.05 was considered significant.

## 3. Results

### 3.1. Plasma Chemerin in SIRS/Sepsis Patients and Controls

**Figure 1:**
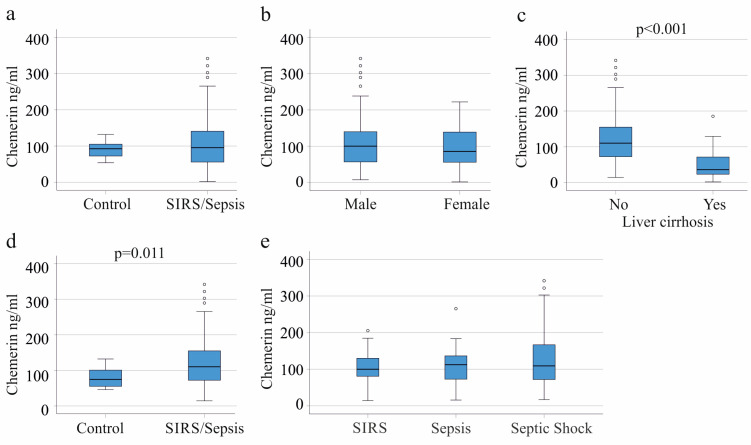
Chemerin in plasma of controls, SIRS/sepsis patients, and SIRS/sepsis patients with liver cirrhosis. (**a**) Plasma chemerin levels of controls and SIRS/sepsis patients; (**b**) plasma chemerin of male and female patients; (**c**) plasma chemerin of severely ill patients with and without liver cirrhosis; (**d**) plasma chemerin of controls and SIRS/sepsis patients after exclusion of patients with liver cirrhosis; (**e**) plasma chemerin of patients with SIRS, sepsis and septic shock (after exclusion of patients with liver cirrhosis). The small circles above the boxes mark outliers. The *p*-values for significant differences are shown in the respective figures.

The patient cohort is described in Table 1. The median age of the patients was 59 years, and a third were women. Plasma chemerin levels of the 156 SIRS/sepsis patients were similar to the 16 age-matched controls (Figure 1a).

Gender disparity in plasma chemerin levels did not exist in the patient group (Figure 1b). Age was not a confounding factor and the Spearman correlation of plasma chemerin with age was r = −0.133, *p* = 0.097.

Patients with liver cirrhosis have low levels of circulating chemerin [26,27], and plasma chemerin of SIRS/sepsis patients with liver cirrhosis was also low (Figure 1c). Therefore, patients with liver cirrhosis were excluded for further analysis. Characteristics of the remaining cohort are summarized in Table 1. These patients have higher levels of C-reactive protein (CRP) in comparison to the whole cohort (Table 1) in line with CRP being a protein produced by the liver [31,37].

Notably, chemerin was higher in critical illness compared to healthy controls when patients with liver cirrhosis had been excluded (Figure 1d). Plasma chemerin was similar among patients with SIRS, sepsis and septic shock (Figure 1e). 

### 3.2. Plasma Chemerin in SIRS/Sepsis Patients with Different Underlying Diseases including COVID-19

**Figure 2:**
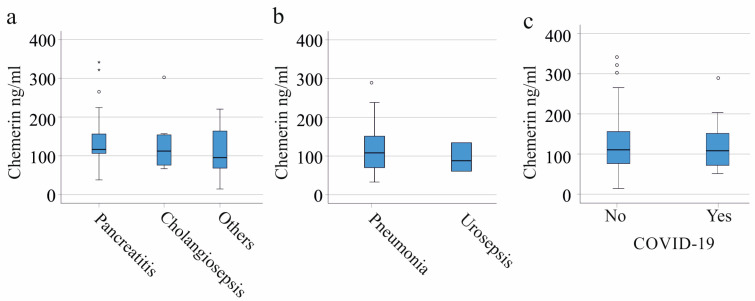
Chemerin in plasma of critically ill patients (patients with liver cirrhosis were excluded) stratified for underlying diseases and causes of severe illness. (**a**) Plasma chemerin levels of SIRS/sepsis patients with different underlying diseases (pancreatitis: 31 patients, cholangiosepsis: 9 patients, others: 29 patients); (**b**) plasma chemerin of SIRS/sepsis patients with pneumonia (41 patients) and urosepsis (14 patients); (**c**) plasma chemerin of SIRS/sepsis patients without and with (21 patients) SARS-CoV-2 infection. There were no significant differences among the groups. The small circles and asterisks above the boxes mark outliers.

Common underlying diseases of the severely ill patients were liver cirrhosis (32 patients), pancreatitis (31 patients) and cholangiosepsis (9 patients). Patients with other diseases such as metabolic acidosis or cancers were rare and were grouped together (29 patients). Plasma chemerin levels did not differ between these three latter groups (Figure 2a). Pulmonary (41 patients) and urinary tract infections (14 patients) were major causes of critical illness. Plasma chemerin was similar among these patients (Figure 2b). 

Twenty-one patients were infected with SARS-CoV-2. This subgroup had similar leukocyte count and CRP levels in comparison to the entire cohort, whereas procalcitonin levels were reduced (Table 1). Plasma chemerin levels were similar between severely ill patients with and without COVID-19 (Figure 2c). It is worth noting that SIRS/sepsis patients infected with SARS-CoV-2 had higher plasma chemerin levels compared to the healthy controls (*p* = 0.037). 

### 3.3. Plasma Chemerin in Relation to Interventions and Vasopressor Therapy

Plasma chemerin was similar between the 38 patients on dialysis and the 86 patients without extracorporeal organ support (Table 2). Mechanical ventilation of 75 patients was not related to a change in plasma chemerin levels (Table 2). Seventy-four of the patients with sepsis were under vasopressor therapy, which was not related to altered plasma chemerin levels (Table 2). 

### 3.4. Plasma Chemerin in Relation to Inflammation Markers

Plasma chemerin positively correlated with leukocyte count (r = 0.164, *p* = 0.041) and CRP (r = 0.516, *p* < 0.001) in the entire patient cohort. Procalcitonin levels were not associated with plasma chemerin levels (r = −0.041, *p* = 0.612). The correlation of chemerin with CRP remained significant after the exclusion of patients with liver cirrhosis (r = 0.384, *p* < 0.001).

In the subcohort of COVID-19 patients plasma chemerin did not correlate with CRP (r = 0.219, *p* = 0.339), procalcitonin (r = 0.112, *p* = 0.630) or IL-6 (r = 0.165, *p* = 0.475). Chemerin was positively correlated with leukocyte count (r = 0.491, *p* = 0.024). 

### 3.5. Plasma Chemerin in Relation to Gram-Negative and Gram-Positive Bacteria

**Figure 3:**
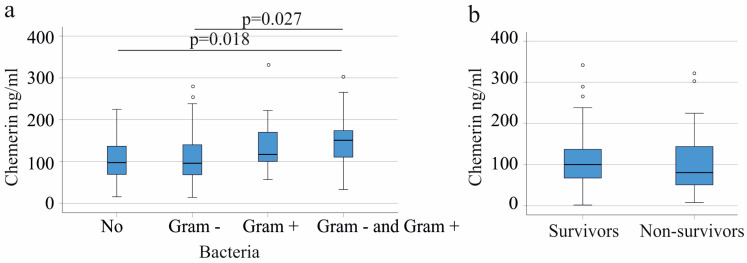
Chemerin in plasma of critically ill patients according to bacterial infection and association of plasma chemerin with survival. (**a**) Plasma chemerin levels of SIRS/sepsis patients (patients with liver cirrhosis were excluded) with no bacteria detected (No), infected with Gram-negative bacteria (gram −), infected with Gram-positive bacteria (gram +) or both types of bacteria. The *p*-values for significant differences are shown in the figure; (**b**) plasma chemerin of all patients in relation to survival. The small circles above the boxes mark outliers.

Plasma chemerin levels of the 44 patients where no infectious agent could be detected in blood were comparable to the 52 patients infected by Gram-negative bacteria (Escherichia coli: 30 patients and Enterococcus faecalis: 30 patients) and the 15 patients infected with Gram-positive bacteria (Staphylococcus aureus: 3 patients and Staphylococcus epidermidis: 12 patients). Median chemerin levels did not differ between the 20 patients infected only with Escherichia coli (90.1 (16.9–289.3) ng/mL) and the 21 patients infected only with Enterococcus faecalis (113.5 (14.3–321.7) ng/mL) (*p* = 0.285). Patients infected with both Gram-negative bacteria had similar chemerin levels compared to patients with monoinfection. Chemerin levels of Staphylococcus aureus-infected patients was 114.1 (94.2–169.7) and of Staphylococcus epidermidis-infected patients was 100.0 (14.3–312.7) (*p* = 0.945). There were no patients who were infected with both Gram-positive bacteria. The 14 patients who had Gram-negative as well as Gram-positive bacteria in their blood had higher plasma chemerin levels in comparison to non-infected patients and patients with Gram-negative infections (Figure 3a). Associations of plasma chemerin with Gram-positive infections were not identified in the subgroup of patients with liver cirrhosis (*p* > 0.05)

Leukocyte number (*p* = 0.490), procalcitonin (*p* = 0.215) and CRP (*p* = 0.564) were not different between patients with no infections, Gram-negative or Gram-positive infections and patients with Gram-negative/Gram-positive infections. 

### 3.6. Plasma Chemerin in Relation to Survival

Thirty-eight of the 156 patients died. Plasma chemerin at the time of admission did not differ between survivors and non-survivors (Figure 3b). Mortality did not differ among COVID-19 (9 of the 23 patients died) and non-COVID-19 (29 of the 132 patients died) patients (*p* > 0.05). In both cohorts, survivors and non-survivors had comparable plasma chemerin levels (*p* = 0.124 and 0.477, respectively). The exclusion of patients with liver cirrhosis did not change these findings. In this subgroup, the 25 patients who died had plasma chemerin levels comparable with the 99 patients who survived (*p* = 0.782). 

It is of clinical relevance that chemerin was increased in patients with Gram-positive infections (Figure 3a). In the subgroup of patients with Gram-positive infections, plasma chemerin did not differ between survivors and non-survivors (*p* = 0.328). 

## 4. Discussion

The current analysis revealed higher plasma chemerin in severely ill patients compared to healthy controls. Further, an increase in chemerin levels occurred in patients infected with Gram-positive bacteria. Our data suggest that plasma chemerin is an emerging candidate for early diagnosis of Gram-positive infection in severely ill patients.

There is strong evidence for higher circulating chemerin levels in patients with inflammatory diseases [17,38]. Elevated serum chemerin levels in sepsis patients in comparison to healthy controls have been described before [18,19]. In our patient cohort, and in accordance with earlier investigations having shown low circulating chemerin levels in patients with viral and alcoholic liver cirrhosis [26,27], we found plasma chemerin to be strongly reduced in patients with liver cirrhosis. The cohorts of sepsis patients described by Horn et al. and Karampela et al. as showing higher chemerin in sepsis did not include patients with liver cirrhosis [18,19]. The exclusion of patients with underlying liver cirrhosis from our cohort revealed that plasma chemerin was about 1.47-fold higher in patients with sepsis and this is comparable to the 1.69-fold increase described earlier [19].

Systemic chemerin was positively correlated with leukocyte count and CRP in our cohort. Associations of systemic chemerin with CRP were observed in different patient cohorts such as rheumatoid arthritis, systemic sclerosis and colorectal cancer [39,40,41]. Hence, as has been shown for CRP [42], higher plasma chemerin is not a specific sepsis marker.

In our patient cohort, plasma chemerin was not related to interventions such as mechanical ventilation and dialysis or vasopressor treatment. Associations with survival were not observed in the current cohort, thus showing that chemerin is not a marker of disease severity or outcome. This finding contradicts earlier studies where patients who died had increased serum chemerin levels [18]. Higher chemerin was also found associated with disease severity and death in COVID-19 [22].

Mortality for COVID-19 patients was about 33% and was higher compared to patients not infected by this virus with a mortality of 18%. This difference did, however, not reach significance. Higher mortality for COVID-19 patients has been reported and significant differences in mortality need validation in larger cohorts [43].

Whether systemic chemerin is changed in SARS-CoV-2 infection has still not been clarified. In COVID-19 patients, serum chemerin was found to be reduced as well as increased in comparison to healthy controls [21,22]. In line with this latter study, our analysis detected higher plasma chemerin levels in patients with severe COVID-19 in comparison to healthy controls. However, in our critically ill patient cohort, patients with and without COVID-19 had similar plasma chemerin levels showing that circulating concentrations of chemerin are raised in patients with severe diseases and are not specifically increased in COVID-19.

*Staphylococcus aureus* and *Staphylococcus epidermidis* are among the most common human pathogens implicated in sepsis [44]. The cysteine protease staphopain B secreted by *Staphylococcus aureus* was shown to process chemerin, thereby producing a chemerin variant acting as an immune cell chemoattractant [45]. *Staphylococcus epidermidis* expresses a cysteine protease similar to staphopain B [46], but so far there is no experimental proof for the role of this protease in chemerin cleavage. Interestingly, the chemerin-derived peptide Val^66^-Pro^85^ was found to restrict the growth of methicillin-resistant *Staphylococcus aureus* [47]. Our analysis showed that patients infected with the Gram-positive bacteria *Staphylococcus aureus* and/or *Staphylococcus epidermidis* have higher plasma chemerin in comparison to non-infected patients, as well as in comparison to patients with Gram-negative bacteria. Co-infection of patients with Gram-negative and Gram-positive bacteria was related to significantly increased chemerin levels in comparison to non-infected patients and patients infected with Gram-negative bacteria. There is experimental evidence that the host response to Gram-positive and Gram-negative organisms greatly differs [48] and chemerin may thus become an early biomarker for Gram-positive infections and Gram-positive SIRS/sepsis. 

Blood cultures can take up to 3 days to detect bacterial growth and it takes even longer to identify the exact pathogen [35]. Therefore, antibiotic selection in early sepsis is empirical [49,50]. Chemerin may become a biomarker for Gram-positive sepsis, allowing a more rational antibiotic therapy. This may also help to avoid bacterial resistance to antibiotics. The overall cost of therapy can be reduced with the use of appropriate antibiotics. 

Currently, we cannot explain why chemerin levels in Gram-positive SIRS/sepsis patients are high. Leukocyte count, procalcitonin and CRP levels did not differ between patients without a positive blood culture, patients with Gram-negative or Gram-positive bacteria and patients infected with both types of bacteria. This indicates that higher chemerin is not related to differences in disease severity among these subcohorts.

Plasma chemerin was not yet found to increase in patients with Gram-positive infections and liver cirrhosis, possibly due to the small cohort. By expansion of the cohort size, higher chemerin in Gram-positive infection may be also detected in liver cirrhosis. The severity of the liver disease is negatively correlated with systemic chemerin [26,51], and this needs to be accounted for.

Plasma chemerin of surviving and non-surviving patients infected with Gram-positive bacteria was, however, similar excluding a survival advantage of those with higher circulating chemerin.

Lipopolysaccharides are toxins of Gram-negative bacteria and were strong inducers of chemerin in adipocytes [15,52]. In primary human hepatocytes, which have a similarly high expression of chemerin as adipocytes, no such regulation has been described [53]. Plasma chemerin levels were not increased in severely ill patients infected with gram-negative bacteria in comparison to patients where no infectious agent could be identified. Hence, lipopolysaccharide seems to have no effect on circulating chemerin levels in critically ill patients.

Frequent causes of sepsis in our patient cohort were pancreatitis and cholangiosepsis. Plasma chemerin was similar between these groups. Higher chemerin has been described in patients with chronic pancreatitis in comparison to healthy controls [54,55]. Current observation indicates that higher systemic chemerin in pancreatitis is not specific to this disease entity and is more likely a marker for a severe illness.

This study has limitations. First, most patients enrolled in this single-center study originated from Germany and results may not be valid for other ethnicities. The sub-cohorts with SARS-CoV-2 infection and liver cirrhosis were rather small, and this limits statistical power. For this reason, the high mortality among COVID-19 patients already shown [43] could not be confirmed. This is an observational study and does not provide functional data on the increase of plasma chemerin in Gram-positive infection.

## 5. Conclusions

Current analysis revealed plasma chemerin as a possible early biomarker for (co)-infection with Gram-positive bacteria in patients with sepsis. Future research in larger cohorts has to evaluate the clinical utility of chemerin.

## Figures and Tables

**Table 1:** Characteristics of the whole cohort, the cohort after excluding patients with liver cirrhosis and of COVID-19 patients (after excluding patients with liver cirrhosis) ^1^.

Parameters	Whole Cohort	Patients without Liver Cirrhosis	COVID-19 Patients without Liver Cirrhosis
Males/Females	109/47	86/38	15/6
Age (years)	59 (21–93)	57 (21–88)	63 (29–80)
C-reactive Protein mg/L	157 (12–697) ***	183 (35–597) ***	156 (44–472)
Procalcitonin ng/mL	1.15 (0.05–270) *	1.17 (0.06–270.00)	0.57 (0.08–65.40) *
Leukocytes n × 10^9^/L	10.31 (0.06–1586.00)	10.35 (2.16–37.38)	9.62 (2.78–18.47)
IL-6 pg/mL	n.d.	n.d.	74.1 (15.7–8679.0)
SIRS/Sepsis/Septic Shock	37/40/79	27/34/63	0/2/19
Dialysis/Ventilation	54/95	38/75	9/21
Vasopressor Therapy	95	74	19
Pancreatitis/Cholangiosepsis	32/13	31/9	0/0
Pneumonia/Urosepsis	54 **/15	41/14	21 **/0

^1^ The subcohorts were compared to the whole cohort and variables, which differed significantly from the whole cohort were labeled with identical symbols. The respective *p*-values are * *p* < 0.05, ** *p* < 0.01, *** *p* < 0.001; not determined, n.d.

**Table 2:** Plasma chemerin levels of patients on dialysis, ventilation or vasopressor therapy in comparison to patients without these interventions/therapy.

Intervention/Drug	No	Yes	*p*-Value
Dialysis	110.7 (14.3–289.3) ng/mL	101.9 (16.9–289.3) ng/mL	0.888
Ventilation	100.0 (14.3–265.4) ng/mL	112.2 (16.90–341.6) ng/mL	0.172
Catecholamine	106.3 (15.7–205.4) ng/mL	113.3 (16.9–341.6) ng/mL	0.222

## Data Availability

Data supporting reported results can be obtained from the corresponding author.
